# Occupational Low-Dose Radiation Affects the Expression of Immune Checkpoint of Medical Radiologists

**DOI:** 10.3390/ijerph19127105

**Published:** 2022-06-09

**Authors:** Chen Wang, Changfu Hao, Kai Dai, Yuzheng Li, Jie Jiao, Zhuoya Niu, Xiao Xu, Xuedan Deng, Jing He, Wu Yao

**Affiliations:** 1 Department of Occupational Health and Environmental Health, College of Public Health, Zhengzhou University, Zhengzhou 450001, China; wang2426500823@163.com (C.W.); haochangfu@126.com (C.H.); daikai0301@163.com (K.D.); niuzya@163.com (Z.N.); xxiao1995@hotmail.com (X.X.); dxd690805@163.com (X.D.); 15932291792@163.com (J.H.); 2 Henan Institute of Occupational Medicine, Zhengzhou 450001, China; 18838008048@163.com (Y.L.); jiaojie66@126.com (J.J.)

**Keywords:** low-dose radiation, radiation workers, T lymphocytes, immune checkpoint, CTLA-4, TIM-3

## Abstract

The purpose of this study was to investigate the expression of immune checkpoint cytotoxic T-lymphocyte-associated protein 4 (CTLA-4) and T cell immunoglobulin and mucin domain 3 (TIM-3) in the peripheral blood T lymphocytes of medical radiologists. The study incorporated 100 male medical radiologists and 107 male healthy controls. The expressions of CTLA-4 and TIM-3 among CD4+ and CD8+ lymphocytes were detected by flow cytometry. The expression levels of CTLA-4 and TIM-3 in the CD4+T cells of radiation workers were lower than those of healthy controls (*p* < 0.05). Correlation analysis showed that the CD8+CTLA-4 expression level was significantly positively correlated with individual cumulative dose (*r_s_* = 0.260, *p* = 0.001, <0.05), while the expression level of CD8+TIM-3 was negatively correlated (*r_s_* = −0.180, *p* = 0.027, <0.05). Low-dose radiation exposure affects the expression of CTLA-4 and TIM-3 in human peripheral blood T lymphocytes. Future studies need to focus on exploring the mechanisms by which CTLA-4 and TIM-3 expression changes in response to low-dose radiation exposure.

## 1. Introduction

With the advancement and development of medical services and nuclear power projects, the number of institutions and people exposed to and engaged in radioactive work has increased year by year. Between 1987 and 2006, the number of people in the United States exposed to ionizing radiation from medical sources increased by 300% [1]. It is estimated that current exposure from medical causes is equivalent to that from natural background radiation [2]. Therefore, the health effects of exposure to low-dose radiation are of particular concern. Although the international commission on radiological protection recommended an annual average effective dose of 20 mSv for five years, and further stipulated that the effective dose should not exceed 50 mSv in any given year, radiation workers are still at high risk [3]. Many studies have shown that ionizing radiation can affect the health of radiation workers. Chronic exposure to ionizing radiation has been found to lead to an increased risk of thyroid disease among radiotherapy professionals [4]. Cardiovascular disease may also be associated with low doses of ionizing radiation [5]. Exposure to ionizing radiation also increases the risk of lens opacity [6]. In addition, there is increasing evidence that low-dose radiation can affect the immune system [7], inducing both innate and adaptive immunity [8]. Therefore, it is very momentous to find sensitive biomarkers to detect the effects of low-dose radiation on radiation workers in time. At present, chromosome aberration rate analysis, micronucleus analysis, and fluorescence in situ hybridization translocation analysis are commonly used to detect radiation damage [9]. Among them, chromosome aberration rate analysis is regarded as the “gold standard” of radiation biological dosimetry [10,11]. However, this method is labor-intensive and time consuming, requiring 72 to 96 h for dose estimation, and therefore additional studies on other radiation-sensitive indicators should be increased in order to discover new and simple biomarkers of radiation damage.

Lymphocyte is one of the most sensitive cell populations to ionizing radiation [12] and is an important reference index for the early diagnosis of radiation injury and the estimation of exposure dose. Different lymphocyte subsets have different radiosensitivity, but research shows that CD8^+^T cells and CD4^+^T cells seem to be the most sensitive to radiation [13].

Immune checkpoints, also known as “co-inhibitory receptors”, are a group of inhibitory or stimulatory molecules expressed on immune cells, antigen presenting cells, tumor cells, or other types of immune cells that mediate the processes of the adaptive immune system, especially T cells and the natural immune system [14]. Immune checkpoint molecules play an important role in the regulation of autoimmune diseases, chronic viral infections, and the treatment of tumors [15,16], and also inhibit the activation and proliferation of lymphocytes [17]. Under normal physiological conditions, immune checkpoints are essential for maintaining self-tolerance (that is, preventing autoimmunity), and they also protect tissue from damage when the immune system responds to a pathogenic infection [18]. CTLA-4 (cytotoxic T-lymphocyte-associated protein 4) and TIM-3 (T cell immunoglobulin and mucin domain 3) are the more interesting examples of immune checkpoint molecules [19]. CTLA-4 is a negative immunoregulatory factor, belonging to the CD28 immunoglobulin subfamily, which is constitutively expressed on regulatory T (Treg) cells and upregulated on activated T cells, and it inhibits T cell activation by binding to the ligand CD80/CD86 [20,21,22]. TIM-3, a member of the TIMs family, is also a negative regulator involved in promoting immune tolerance [23]. A large number of studies have shown that they inhibit the development of autoimmunity by inhibiting the activation of T cells by mediating immune responses [19,20], which can reflect the immune state of the body to a certain extent.

In recent years, a large number of studies have confirmed that immune checkpoints play an important role in maintaining immune homeostasis., and they also have potential biomarker and intervention target value in tumors and other diseases. However, their expression characteristics, functions, and significances in ionizing radiation have not been reported yet. Therefore, this study investigated the expression levels and characteristics of peripheral blood T lymphocytes CD3+T, CD4+T, and CD8+T, and their immune checkpoints CTLA-4 and TIM-3 in medical radiologists exposed to long-term low-dose ionizing radiation, and we analyzed the correlation with low-dose ionizing radiation exposure dose and biological effects. Explored the potential of immune checkpoints as biomarkers and immune status monitoring indicators in occupational populations exposed to low-dose ionizing radiation. Thus, this study provided new ideas and references for improving the health monitoring of radiation workers and strengthening radiation protection.

## 2. Materials and Methods

### 2.1. Study Population and Ethics Statement

Radiation worker: This study recruited 100 male radiation workers, aged 22 to 64, from hospitals in Zhengzhou, capital of Henan Province, China. Participants’ occupational exposure to ionizing radiation ranged from 1 to 43 years. All participants were interviewed by professional interviewers and completed a questionnaire about demographics, smoking and alcohol history, occupational history, hours of work, and occupational and personal medical history. Exclusion criteria included autoimmune disease and previous occupational history.

Healthy control group: In this study, 107 healthy male subjects, whose age matched with the radiation exposure group, were selected from healthy subjects in the health examination center of the hospital during the same period.

This study was conducted in Henan Institute of Occupational Disease Prevention and Control. Approved by the ethics committee of the institute, about 2 mL peripheral blood was collected from each physical examination subject with vacuum tube after the informed consent of the patients.

### 2.2. Individual Monitoring of Occupational External Exposure

According to the National Standard of the People’s Republic of China (GBZ 128-2016) formulated by the National Health and Family Planning Commission of the People’s Republic of China, the annual human effective dose is monitored using a thermoluminescence dosimeter (PTW-Freiburg, Freiburg im Breisgau, Germany). An individual’s external radiation dose was measured four times a year for 3 months. After removing distorted data, the annual effective dose was calculated as follows:

$$H_{p}\;(10)=(\bar{x}-\bar{x_{0}})\times Cf$$

where *Hp* (10), $\bar{x},\bar{x_{0}}$, and *Cf* indicate individual penetrating dose equivalent (mSv), the mean of the background reading (mGy), the mean of the measured value (mGy), and the calibration coefficient of the thermoluminescent dosimeter (mSv), respectively.

### 2.3. Sample Preparation

Venous blood samples were collected using disposable sterile needles and collected into an EDTA anticoagulant tube. The blood samples were coded and stored at 4 °C and transported to the laboratory for testing. Then, 150 μL human peripheral blood was taken, mixed with 1× Lysing Buffer (BD Biosciences, San Jose, CA, USA), and lysed twice until no obvious RBC precipitate was found. White blood cells were obtained by re-suspension with PBS and centrifugation. The cells were suspended with 100 μL FBS for subsequent flow cytometry antibody staining.

### 2.4. Flow Cytometry

Firstly, 10^6^ cells from each sample were suspended on 100 μL FBS, and appropriate volumes of BB515-conjugated CD3, PerCP-cy-conjugated CD4, BV605-conjugated CD8, and APC-conjugated TIM-3 were added. Another tube of sample was added with corresponding volumes of BB515-conjugated CD3, PerCP-cy-conjugated CD4, BV605-conjugated CD8 antibodies, and matched fluorescence-labeled isotypes of APC-conjugated TIM-3. Then, they were vortexed for 3 s, and incubated at 4 °C for 30 min in the dark. Then, added 1 mL FBS, 300 g, 4 °C, 5 min, and discarded the supernatant. The cells were suspended with 300 μL Cytofix/Cytoperm Soln Kit for 3 s, and then incubated at 4 °C for 20 min in the dark. After that, 1 mL 1× washing buffer was added, 300 g, 4 °C, 5 min, and the supernatant was discarded. The cells were suspended with 100 μL 1 × washing buffer, followed by the addition of appropriate volume of PE-conjugated CTLA-4, and the corresponding volume of PE-conjugated CTLA-4-matched fluorescent-labeled negative control was added into the same type of control, then the cells were incubated by vortex for 3 s and sheltered from light at 4 °C for 30 min. After that, 1 mL 1× washing buffer was added, 300 g, 4 °C, 5 min, and the supernatant was discarded. After repeated cleaning for two times, the cells were fixed and suspended with 500 μL 4% paraformaldehyde and stored overnight at 4 °C for machine operation.

**Figure 1:**
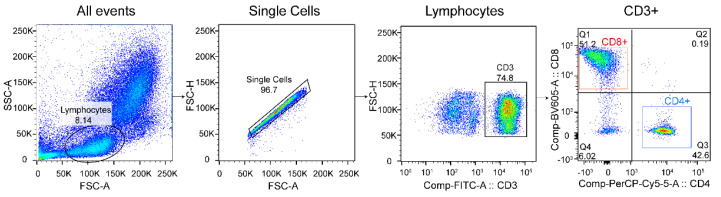
Example lineage gating strategy using pseudocolor dot plots. Figure generated using Flow Jo.

**Figure 2:**
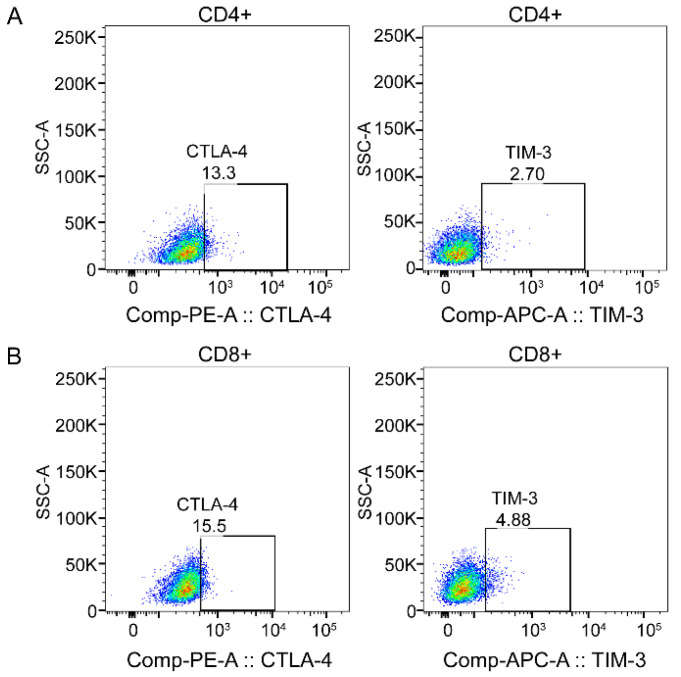
Example functional marker gating strategy for CD4+T and CD8+T lymphocytes. (**A**) Distribution of CTLA-4 and TIM-3 on CD8+T cells; (**B**) distribution of CTLA-4 and TIM-3 on CD4+T cells. Figure generated using Flow Jo.

The blank sample tube was used to adjust the channel voltage of the instrument before testing samples, and the monochromatic compensation microsphere (BD Biosciences, San Jose, CA, USA) was used to set the compensation. Then, 2–3 × 10^4^ target cells were collected in each sample using a three-laser flow cytometry (BD FACS Canto) and subsequent analysis was performed using Flow Jo software. The lymphocyte, single cells, CD3+T, CD4+T, and CD8+T lymphocyte subsets were then gated by drawing regions (Figure 1). The immune checkpoints CTLA-4 and TIM-3 were gated in CD4+T cells and CD8+T cells, respectively, according to negative control (Figure 2).

### 2.5. Statistical Analysis

Statistical analysis was performed using SPSS version 25.0 (IBM, Armonk, NY, USA). Descriptive data in this study were presented in the form of mean, median, range, standard deviation, and percentage. The *χ*^2^ test was used to evaluate the differences in the distribution of categorical variables between groups. The Mann–Whitney U test or a generalized linear model was used to assess inter-group differences in continuous measurement data. Spearman correlation coefficients were calculated to evaluate the association between relevant parameter. All statistical tests were based on a 2-sided probability, with a significance level of 0.05.

## 3. Results

### 3.1. Demographic Characteristics of the Study Population

The 100 radiation workers and 107 healthy controls included in the study were divided into two groups at 38 years of age respectively; the body mass index (BMI) of the radiation exposure group and the healthy control group were divided into four groups according to the Chinese population classification standard: low body weight (BMI < 18.5), normal body weight (BMI 18.5 to 23.9), overweight (BMI 23.9 to 27.9), and obesity (BMI > 27.9). The results showed that there were no significant differences in age and BMI between the two groups (*p* > 0.05). There were statistically significant differences in current smoking (*χ*^2^ = 32.749, *p* < 0.001) and current alcohol consumption (*χ*^2^ = 15.503, *p* < 0.001) between the two groups. The average length of service of radiation workers was 13.3 ± 1.1 years. Radiation workers were divided into four groups according to their work at the time of examination, including diagnostic radiology (n = 28), radiotherapy (n = 12), nuclear medicine (n = 5), and interventional radiology (n = 55), the annual average monitoring dose of each group was replaced by the annual average dose value of the same job in the same year, and the cumulative dose was calculated by combining individual years of service. The average cumulative dose of radiation workers in this study was 5.70 ± 0.48 mSv. Specific results are shown in Table 1.

### 3.2. Peripheral Blood Results Analysis

By comparing the results of peripheral blood imaging, there were no statistical differences in WBC (Z = −1.381, *p* = 0.167) and LY (Z = −1.427, *p* = 0.154) between the two groups, while the RBC (*p* = 0.01, <0.05), Hb (*p* < 0.001), and PLT (*p* = 0.003, <0.05) of the radiation workers were lower than those in control group, and the difference was statistically significant. After adjusting for factors such as age, BMI, and smoking and drinking using generalized linear models, the RBC (*p* = 0.01, <0.05) and Hb (*p* < 0.001) of the radiation workers were lower than those in control group (Table 2).

### 3.3. The Relative Expression Levels of CD3+T, CD4+T, and CD8+T Cells and TIM-3 and CTLA-4

The percentage of CD8+T cells in radiation workers was significantly higher than that in healthy controls, and the difference was statistically significant (Z = −2.513, *p* = 0.012, <0.05). The percentages of CD4+CTLA-4+ cells (Z = −4.680, *p* = <0.001) and CD4+TIM-3+cells (Z = −2.634, *p* = 0.008, <0.05) in radiation workers were significantly lower than those in healthy controls. However, there was no statistical difference in the percentage of CD3+T, CD4+T, CD8+CTLA-4+, and CD8+TIM3+cells between the two groups (*p* > 0.05) (Table 3).

### 3.4. The Relationship between Demographic Characteristics and Percentage of CD4+T and CD8+T Cells and Expression of CTLA-4 and TIM-3

After adjusting for age, smoking, alcohol consumption, and BMI, the effects of demographic characteristics such as age, BMI, smoking, and alcohol consumption on the percentage of CD4+T and CD8+T cells and the expression of CTLA-4 and TIM-3 were further analyzed by using the generalized linear model.

As shown in Table 4, there was no statistically significant difference in the percentages of CD4+T and CD8+T cells between the two groups at different ages (*p* > 0.05), but the differences between the BMI < 18.5 and BMI > 27 levels were statistically significant (*p* < 0.05). In the smokers, the percentage of CD8+T cells in the radiation workers was higher than in the healthy controls (*p* < 0.05), as well as in the current abstainers. Intragroup analysis showed that smoking status had an effect on the percentages of CD4+T and CD8+T cells in healthy controls (*p* < 0.05), while there were no statistically significant differences among age, alcohol consumption, and BMI (*p* > 0.05).

As shown in Table 5, there was no statistically significant difference in the expression of CTLA-4 and TIM-3 in CD8+Tcells between the two groups in age and alcohol consumption (*p* > 0.05), but the differences between BMI were statistically significant (*p* < 0.05). The differences in TIM-3 expression of CD8+Tcells between the two groups were statistically significant (*p* < 0.05), and the expression in radiation workers was lower than in healthy controls. Among radiologists, smoking and BMI affected the expression of TIM-3 in CD8+T cells (*p* < 0.05).

As shown in Table 6, the expression of CTLA-4 in CD4+T cells of radiation workers was lower than that of healthy controls at the levels of age, BMI, smoking, and alcohol consumption (*p* < 0.05), and the expression of TIM-3 in CD4+T cells was statistically different between the two groups (*p* < 0.05). Intragroup analysis showed that age affected the expression of CTLA-4 in the CD4+T cells of the control group (*p* < 0.05), smoking affected the expression of TIM-3 in the CD4+T cells of radiation workers (*p* < 0.05), and BMI affected the expression of TIM-3 in the CD4+T cells of radiation workers (*p* < 0.05).

### 3.5. Relationship between Cumulative Exposure Dose and CTLA-4 and TIM-3 Expression Levels in Peripheral Blood Lymphocytes of Radiation Workers

As shown in Table 7 and Table 8, radiologists were divided into four subgroups according to the quartile of cumulative dose exposure. The doses from low to high were a 0–1.73 mSv group, 1.73–4.33 mSv group, 4.33–8.65 mSv group, and >8.65 mSv group. We took the 0–1.73 mSv group as the reference group after adjusting for age, smoking, alcohol consumption, and BMI, and the effects of a cumulative exposure dose on the expression of CTLA-4 and TIM-3 were further analyzed by using the generalized linear model. In terms of CD8+CTLA-4, the CD8+CTLA-4 expression level in the >8.65 mSv group was 9.66 (5.25, 16.55)%, which was significantly higher than that in the 0–1.73 mSv group (6.25 (2.90, 8.70)% *p* < 0.05). While for the CD8+TIM-3 expression level, compared to the 0–1.73 mSv group, the expression of the other three groups was not significantly different (*p* > 0.05). In addition, for the CD4+CTLA-4 expression level, compared to the 0–1.73 mSv group, the expression of the other three groups was not significantly different (*p* > 0.05). The CD4+TIM-3 expression level in the 0–1.73 mSv group (4.21 (3.53, 7.20)%) was significantly lower than that in the 4.33–8.65 mSv and >8.65 mSv groups (4.36 (3.65, 5.30)% and 4.29 (3.02, 5.79)%, respectively; *p* < 0.05).

To evaluate the relationship between CTLA-4 and TIM-3 levels in peripheral blood lymphocytes and cumulative dose, the correlation analysis was performed. Results showed a significant positive correlation between the CD8+CTLA-4 expression level and individual cumulative dose (*r_s_* = 0.260, *p* = 0.001, <0.05). This funding revealed that CD8+CTLA-4 levels increased with an increasing cumulative radiation dose. By contrast, the CD8+TIM-3 expression level was significantly negatively correlated with individual cumulative dose (*r_s_* = −0.180, *p* = 0.027, <0.05), which suggested that CD8+TIM-3 levels decreased with an increasing cumulative radiation dose.

## 4. Discussion

To date, the health effects of long-term exposure to low-dose radiation remain a subject of significant scientific concern. Medical workers are a common study group among professionals exposed to radiation for a long period of time and are subject to regular medical monitoring and dosimetry [24], of which peripheral blood imaging is an important health monitoring indicator. By analyzing the blood indicators of the subjects, we found that the red blood cells count (RBC) and hemoglobin (Hb) counts of the radiation workers were significantly lower than those of the healthy control group (*p* < 0.05), which was consistent with the observation of Qing-Zeng Qian et al. on the radiologists in Tangshan, China [25]. It indicates that long-term exposure to low-dose radiation has obviously affected the health of the exposed group.

The immune system is one of the important defense mechanisms against various environmental risk factors and is greatly affected by ionizing radiation. Numerous experimental and epidemiological studies clearly demonstrate the immunosuppressive effects of high doses of radiation [26,27]. However, the effects of low-dose radiation on the immune system remain highly controversial [12]. A review by Katalin et al. described the immunosuppressive effects of low-dose radiation [28], while other studies described the stimulative effects of low-dose radiation, including stimulating growth rates, down-regulating tumor incidence [29], and stimulating the immune system [30]. Immune checkpoint molecules play an important role in maintaining immune homeostasis, and after T cell activation, inhibitory receptors such as CTLA-4 and TIM-3 play a key role in establishing peripheral tolerance and inhibiting T cell proliferation and function [31], and they can balance costimulatory signals and prevent effector T cells from being overactivated, thus avoiding autoimmunity [32]. At present, the detection methods for the expression level of immune checkpoints are relatively mature, including flow cytometry, RT-qPCR, and Western blot, etc. The expression level is reflected in the frequency, mRNA level, and protein level, respectively. The results of the three methods are consistent. However, flow cytometry is faster and easier. Previous studies have shown that CTLA-4 expression levels are elevated in breast cancer patients [33], and the absence of CTLA-4 can cause dysregulation of the immune response [34]. Peng Pu-ji et al. found that TIM-3 was highly expressed in pancreatic cancer tissues [35]. However, Ping Zhang et al. found that TIM-3 expression was down-regulated in colorectal cancer patients [36].

In this study, to explore whether the expression levels of immune checkpoint molecules CTLA-4 and TIM-3 changed after long-term exposure to low-dose radiation, subjects were included without any history of autoimmune disease or related occupational history, and there was no statistical difference in age and BMI between the two groups. We found that CD8^+^T cell levels were significantly higher in radiation workers than in healthy controls (*p* < 0.05), and there was no significant difference in the CD4^+^T cell level between the two groups, which was consistent with the results of Ruchi Pandey et al. [37], possibly because CD8^+^T cells were more sensitive to ionizing radiation than CD4^+^T cells [38]. By analyzing the expression of immune checkpoint molecules on both types of immune cells, we found that CTLA-4 and TIM-3 in the exposed group were down-regulated only on CD4^+^T cells, and there was no significant difference in expression on CD8^+^T cells compared with the control group. Further analysis of the effects of demographic factors such as age, BMI, and smoking and drinking status on CD4^+^T and CD8^+^T cells and the expression levels of CTLA-4 and TIM-3 showed that age, BMI, and smoking status all affected the expression levels of CTLA-4 and TIM-3. We then divided radiologists into four groups according to the quartile of cumulative dose exposure, and analyzed the effects of different levels of radiation dose on the expression levels of CTLA-4 and TIM-3, excluding confounding factors such as age, BMI, and tobacco and alcohol status. The results showed that CD8^+^CTLA-4 expression levels in the 4.33 to 8.65 mSv and >8.65 mSv groups were significantly higher than that in the <1.73 mSv group (*p* < 0.05), which suggested that with the increase of radiation dose, the immune system is stimulated and the expression of CTLA-4 is up-regulated to inhibit the over-activation of T cells. Interestingly, compared with the <1.73 mSv group, the expression level of CD4^+^TIM-3 in the other three groups of radiologists was significantly decreased (*p* < 0.05). Further correlation analysis showed that the CD8^+^T CTLA-4 expression level was significantly positively correlated with individual cumulative dose, while the CD8^+^TIM-3 expression level was significantly negatively correlated with individual cumulative dose, which suggested the particularly complex effects of low-dose radiation on the human immune system; therefore, in vitro irradiation experiments are needed for further study in the future.

The advantage of this study is that the research object not only selects radiology medical workers from different departments, but also includes healthy control population in equal proportion, which makes the comparison more obvious as the sample size reached a certain scale. At the same time, because our data were from a cross-sectional population study, there are limitations in conclusively confirming the association between low-dose radiation exposure and CTLA-4 and TIM-3 expression levels.

## 5. Conclusions

In conclusion, our study shows that occupational low-dose radiation can affect the expression levels of CTLA-4 and TIM-3 in the peripheral blood T lymphocytes of medical radiologists.

## Figures and Tables

**Table 1:** Demographic characteristics of research objects.

Variables		Radiation Workers (n = 100)	Control Group (n = 107)	*χ* ^2^	*p*
Age (year)	≤38	52 (52.0%)	55 (51.4%)	0.007	0.931
	>38	48 (48.0%)	52 (48.6%)		
BMI (kg/m^2^)	<18.5	3 (3%)	1 (0.9%)	4.919	0.171
	18.5~23.9	26 (26.0%)	18 (16.8%)		
	23.9~27.9	55 (55.0%)	62 (57.9%)		
	>27.9	16 (16.0%)	26 (24.3%)		
Current smoking status	Yes	24 (24.0%)	68 (63.6%)	32.749	<0.001
	No	76 (76.0%)	39 (36.4%)		
Current alcohol consumption	Yes	40 (40.0%)	72 (67.3%)	15.503	<0.001
	No	60 (60.0%)	35 (32.7%)		
Length of service (year)		13.3 ± 1.1			
Type of work	interventional radiology	55 (55.0%)			
	radiotherapy	12 (12.0%)			
	diagnostic radiology	28 (28.0%)			
	nuclear medicine	5 (5.0%)			
Cumulative dose (mSv)		5.70 ± 0.48			

**Table 2:** Peripheral blood results analysis.

Variables	Radiation Workers (n = 100)	Control Group (n = 107)	Model 1		Model 2	
			Z	*p*	*χ* ^2^	*p*
WBC (10^9^/L)	6.67 (5.75, 8.24)	7.17 (6.14, 7.82)	−1.381	0.167	0.106	0.745
LY (10^9^/L)	2.23 (1.87, 2.54)	2.38 (1.95, 2.72)	−1.427	0.154	0.001	0.973
RBC (10^12^/L)	5.06 (4.82, 5.28)	5.17 (4.97, 5.39)	−2.570	0.010	6.563	0.010
Hb (g/L)	146.50 (141.00, 152.75)	156.81 (152.00, 163.00)	−7.236	<0.001	40.357	<0.001
PLT (10^9^/L)	238.00 (207.25, 279.75)	267.67 (225.00, 298.00)	−2.996	0.003	2.523	0.112

Abbreviations: Model 1: Mann–Whitney U; Model 2: generalized linear model, adjusted for age, smoking, drinking, and BMI. WBC: white blood cells; LY: lymphocyte; RBC: red blood cell; Hb: hemoglobin; PLT: blood platelet.

**Table 3:** Relative expression levels of CD3+, CD4+, and CD8+ cells and TIM-3 and CTLA-4 in the two groups.

Cells	Radiation Workers (n = 100)	Control Group (n = 107)	Z (*p*)
	M (P25, P75)	M (P25, P75)	
CD3^+^ (%)	61.34 (55.58, 67.90)	63.30 (58.10, 68.80)	−1.574 (0.115)
CD4^−^CD8^+^ (%)	40.55 (33.20, 47.63)	36.70 (29.90, 43.60)	−2.513 (0.012)
CD8^+^CTLA-4^+^ (%)	7.51 (4.17, 10.25)	7.78 (4.84, 10.90)	−0.982 (0.326)
CD8^+^TIM3^+^ (%)	3.45 (2.19, 5.08)	3.53 (2.27, 4.96)	−0.339 (0.735)
CD4^+^CD8^−^ (%)	48.90 (41.53, 57.90)	51.57 (44.40, 59.30)	−1.800 (0.072)
CD4^+^CTLA-4^+^ (%)	5.25 (3.37, 7.26)	7.29 (4.73, 10.70)	−4.680 (<0.001)
CD4^+^TIM3^+^ (%)	4.26 (3.38, 5.84)	5.25 (4.13, 5.99)	−2.634 (0.008)

**Table 4:** Relationship between demographic characteristics and percentage of CD4+ and CD8+cells.

Variables	CD4^+^ (%)					CD8^+^ (%)				
	Radiation Workers (n = 100)		Control Group (n = 107)		*χ*^2^ (*p*) ^a^	Radiation Workers (n = 100)		Control Group (n = 107)		*χ*^2^ (*p*) ^a^
	n	M (P25, P75)	n	M (P25, P75)		n	M (P25, P75)	n	M (P25, P75)	
Age (year)										
≤38	52	48.00 (41.23, 53.60)	55	52.00 (43.70, 58.20)	0.142 (0.706)	52	41.40 (33.73, 47.93)	55	36.70 (32.20, 42.90)	1.720 (0.190)
>38	48	49.13 (42.58, 59.65)	52	51.57 (44.40, 60.48)	0.050 (0.824)	48	39.98 (30.85, 47.40)	52	36.79 (27.70, 43.60)	2.920 (0.087)
*χ*^2^ (*p*) ^b^		2.046 (0.153)		0.018 (0.894)			1.484 (0.223)		0.420 (0.517)	
BMI										
<18.5	3	41.90 (22.40)	1	73.40	25.926 (<0.001)	3	45.00 (42.90)	1	51.8	34.436 (<0.001)
18.5~23.9	26	50.20 (40.48, 60.90)	18	52.50 (44.83, 59.58)	0.065 (0.799)	26	38.65 (28.65, 47.48)	18	36.45 (30.33, 44.90)	0.026 (0.871)
23.9~27.9	55	49.13 (43.10, 58.70)	62	51.10 (43.10, 58.58)	1.194 (0.275)	55	40.46 (32.50, 48.50)	62	36.35 (29.00, 43.60)	3.250 (0.071)
>27.9	16	47.05 (40.35, 50.45)	26	51.95 (44.40, 59.93)	4.081 (0.043)	16	43.70 (37.05, 47.10)	26	36.79 (32.38, 43.28)	4.504 (0.034)
*χ*^2^ (*p*) ^b^		5.322 (0.150)		5.485 (0.140)			4.924 (0.177)		2.859 (0.414)	
Current smoking										
Yes	24	50.35 (40.85, 59.65)	68	54.10 (46.73, 60.88)	2.111 (0.146)	24	40.23 (32.68, 50.48)	68	36.05 (28.40, 43.15)	5.151 (0.023)
No	76	48.30 (41.53, 54.83)	39	48.30 (40.90, 53.70)	1.423 (0.233)	76	40.85 (33.33, 47.38)	39	38.20 (32.80, 45.50)	0.921 (0.337)
*χ*^2^ (*p*) ^b^		0.655 (0.418)		12.261 (<0.001)			0.046 (0.830)		4.075 (0.044)	
Current drinking										
Yes	40	48.90 (41.25, 59.03)	72	51.68 (44.50, 59.90)	0.126 (0.722)	40	40.55 (33.25, 47.85)	72	36.74 (30.25, 43.60)	1.504 (0.220)
No	60	48.75 (42.03, 53.68)	35	51.10 (44.30, 59.10)	0.017 (0.896)	60	40.58 (32.83, 47.63)	35	36.70 (29.50, 42.90)	4.451 (0.035)
χ^2^ (*p*) ^b^		0.006 (0.937)		0.000 (0.999)			0.016 (0.899)		0.655 (0.418)	

Annotation: ^a^ represents the differences in the distribution of CD4^+^ and CD8^+^cells between radiation workers and healthy controls after stratification of variables; ^b^ represents differences in the distribution of CD4^+^ and CD8^+^cells in radiation workers or healthy controls after stratification of variables.

**Table 5:** Relationship between demographic characteristics and the expression of CTLA-4 and TIM-3 in CD8+cells.

Variables	CD8^+^CTLA-4^+^ (%)					CD8^+^TIM-3^+^ (%)				
	Radiation Workers (n = 100)		Control Group (n = 107)		*χ*^2^ (*p*) ^a^	Radiation Workers (n = 100)		Control Group (n = 107)		*χ*^2^ (*p*) ^a^
	n	M (P25, P75)	n	M (P25, P75)		n	M (P25, P75)	n	M (P25, P75)	
Age (year)										
≤38	52	7.18 (4.03, 9.42)	55	7.26 (4.60, 11.10)	0.166 (0.684)	52	3.98 (2.36, 5.48)	55	3.70 (2.81, 4.96)	1.395 (0.237)
>38	48	8.01 (4.38, 10.78)	52	8.06 (5.41, 9.98)	0.671 (0.413)	48	2.99 (2.08, 4.45)	52	3.52 (1.66, 4.95)	0.016 (0.898)
*χ*^2^ (*p*) ^b^		1.405 (0.236)		0.258 (0.612)			0.176 (0.675)		0.480 (0.488)	
BMI										
<18.5	3	8.70 (8.65)	1	1.78	27.725 (<0.001)	3	3.65 (2.20)	1	8.17	37.381 (<0.001)
18.5~23.9	26	7.50 (3.90, 10.53)	18	7.42 (4.61, 9.68)	0.782 (0.376)	26	3.34 (2.20, 5.28)	18	3.73 (2.11, 4.41)	0.005 (0.945)
23.9~27.9	55	8.04 (5.10, 12.60)	62	7.67 (4.91, 11.68)	1.285 (0.257)	55	3.27 (2.04, 4.45)	62	3.59 (2.26, 5.09)	4.973 (0.026)
>27.9	16	3.87 (2.57, 7.81)	26	8.32 (5.23, 10.52)	3.749 (0.053)	16	4.53 (2.54, 8.71)	26	3.52 (2.49, 4.45)	6.661 (0.010)
*χ*^2^ (*p*) ^b^		4.729 (0.193)		3.005 (0.391)			8.803 (0.032)		3.218 (0.359)	
Current smoking										
Yes	24	7.89 (4.66, 9.98)	68	8.87 (4.90, 11.95)	0.400 (0.527)	24	3.94 (2.44, 6.31)	68	4.13 (2.25, 4.96)	6.209 (0.013)
No	76	7.32 (4.03, 10.38)	39	6.88 (4.76, 8.87)	0.640 (0.424)	76	3.26 (2.14, 4.82)	39	3.45 (2.27, 5.01)	2.782 (0.095)
*χ*^2^ (*p*) ^b^		0.394 (0.530)		0.611 (0.434)			6.712 (0.010)		1.045 (0.307)	
Current drinking										
Yes	40	7.98 (4.08, 11.00)	72	7.93 (4.79, 11.35)	0.124 (0.725)	40	3.39 (2.34, 4.83)	72	4.00 (2.44, 5.06)	3.472 (0.062)
No	60	7.26 (4.17, 10.25)	35	6.95 (4.84, 9.04)	3.261 (0.071)	60	3.59 (2.08, 5.57)	35	3.31 (1.92, 4.36)	1.521 (0.217)
*χ*^2^ (*p*) ^b^		0.419 (0.518)		0.351 (0.554)			0.006 (0.937)		0.816 (0.366)	

Annotation: ^a^ represents the differences in the expression of CD8^+^CTLA-4 and CD8^+^TIM-3 between radiation workers and healthy controls after stratification of variables; ^b^ represents differences in the expression of CD8^+^CTLA-4 and CD8^+^TIM-3 in radiation workers or healthy controls after stratification of variables.

**Table 6:** Relationship between demographic characteristics and the expression of CTLA-4 and TIM-3 in CD4+cells.

Variables	CD4^+^CTLA-4^+^ (%)					CD4^+^TIM-3^+^ (%)				
	Radiation Workers (n = 100)		Control Group (n = 107)		*χ*^2^ (*p*) ^a^	Radiation Workers (n = 100)		Control Group (n = 107)		*χ*^2^ (*p*) ^a^
	n	M (P25, P75)	n	M (P25, P75)		n	M (P25, P75)	n	M (P25, P75)	
Age (year)										
≤38	52	5.43 (3.50, 7.26)	55	8.24 (4.93, 12.00)	11.762 (0.001)	52	4.03 (3.17, 5.84)	55	5.26 (4.55, 6.05)	1.357 (0.244)
>38	48	5.06 (2.96, 7.26)	52	6.62 (4.46, 9.27)	5.835 (0.016)	48	4.33 (3.60, 5.86)	52	4.78 (3.64, 5.90)	0.265 (0.607)
*χ*^2^ (*p*) ^b^		0.323 (0.570)		4.768 (0.029)			0.285 (0.593)		1.154 (0.283)	
BMI										
<18.5	3	4.99 (4.46)	1	4.46	31.157 (<0.001)	3	2.86 (2.80)	1	2.86	487.409 (<0.001)
18.5~23.9	26	4.76 (3.42, 7.20)	18	8.08 (4.04, 12.05)	4.079 (0.043)	26	4.26 (3.53, 5.58)	18	5.18 (4.04, 6.32)	1.091 (0.296)
23.9~27.9	55	5.43 (3.78, 6.54)	62	6.78 (4.97, 10.43)	6.382 (0.012)	55	3.87 (3.36, 5.30)	62	4.70 (3.99, 6.06)	0.010 (0.920)
>27.9	16	4.17 (2.09, 7.88)	26	7.93 (4.84, 11.48)	5.132 (0.023)	16	6.06 (3.82, 11.58)	26	5.25 (4.51, 5.81)	10.901 (0.001)
*χ*^2^ (*p*) ^b^		0.393 (0.942)		3.383 (0.336)			10.513 (0.015)		11.640 (0.009)	
Current smoking										
Yes	24	5.67 (3.48, 8.25)	68	6.78 (4.81, 10.58)	2.597 (0.107)	24	5.58 (4.07, 7.74)	68	5.02 (3.89, 6.01)	4.589 (0.032)
No	76	5.03 (3.25, 6.67)	39	7.87 (4.47, 11.00)	17.838 (<0.001)	76	3.83 (3.17, 5.30)	39	5.26 (4.39, 5.99)	0.053 (0.818)
*χ*^2^ (*p*) ^b^		0.820 (0.365)		0.199 (0.656)			7.253 (0.007)		0.406 (0.524)	
Current drinking										
Yes	40	5.52 (3.86, 7.46)	72	6.78 (4.92, 10.68)	4.389 (0.036)	40	4.36 (3.71, 6.13)	72	5.07 (3.95, 6.04)	1.480 (0.224)
No	60	5.09 (2.87, 6.62)	35	7.91 (4.24, 10.90)	14.643 (<0.001)	60	3.79 (3.16, 5.38)	35	5.26 (4.42, 5.90)	0.372 (0.542)
*χ*^2^ (*p*) ^b^		0.644 (0.422)		0.009 (0.924)			0.131 (0.718)		2.131 (0.144)	

Annotation: ^a^ represents the differences in the expression of CD4^+^CTLA-4 and CD4^+^TIM-3 between radiation workers and healthy controls after stratification of variables; ^b^ represents differences in the expression of CD4^+^CTLA-4 and CD4^+^TIM-3 in radiation workers or healthy controls after stratification of variables.

**Table 7:** Relationship between cumulative dose and CTLA-4 and TIM-3 expression levels in CD8+T cell.

Cumulative Dose (mSv)	n	CTLA-4+CD8+T(%)				TIM-3+CD8+T(%)			
		M (P25, P75)	*β* (95% CI)	*χ* ^2^	*p*	M (P25, P75)	*β* (95% CI)	*χ* ^2^	*p*
0~1.73	28	6.25 (2.90, 8.70)	Ref			4.07 (2.52, 5.90)	Ref		
1.73~4.33	23	5.75 (3.41, 8.04)	−0.587 (−3.170, 1.996)	0.198	0.656	3.76 (2.20, 5.04)	−0.895 (−3.112, 1.322)	0.626	0.429
4.33~8.65	27	8.04 (6.45, 10.10)	2.604 (−0.353, 5.561)	2.978	0.084	4.14 (2.36, 5.51)	0.605 (−1.934, 3.143)	0.218	0.641
8.65~	22	9.66 (5.25, 16.55)	6.130 (1.800, 10.459)	7.700	0.006	2.47 (1.70, 3.26)	−2.506 (−6.223, 1.211)	1.746	0.186

Abbreviations: generalized linear model, adjusted for age, smoking, drinking, and BMI.

**Table 8:** Relationship between cumulative dose and CTLA-4 and TIM-3 expression levels in CD4+T cell.

Cumulative Dose (mSv)	n	CTLA-4+CD4+T(%)				TIM-3+CD4+T(%)			
		M (P25, P75)	*β* (95% CI)	*χ* ^2^	*p*	M (P25, P75)	*β* (95% CI)	*χ* ^2^	*p*
0~1.73	28	4.67 (2.90, 6.70)	Ref			4.21 (3.53, 7.20)	Ref		
1.73~4.33	23	4.97 (2.37, 6.72)	−0.245 (−1.807, 1.317)	0.094	0.759	3.76 (3.08, 5.92)	−1.405 (−3.000, 0.190)	2.980	0.084
4.33~8.65	27	5.43 (4.52, 8.01)	1.643 (−0.145, 3.431)	3.243	0.072	4.36 (3.65, 5.30)	−3.130 (−4.957, −1.304)	11.282	0.001
8.65~	22	5.32 (3.34, 7.52)	1.770 (−0.848, 4.388)	1.755	0.185	4.29 (3.02, 5.79)	−4.150 (−6.825, −1.476)	9.252	0.002

Abbreviations: generalized linear model, adjusted for age, smoking, drinking, and BMI.
